# Migration of a fractured inferior vena cava filter strut to the right ventricle of the heart: a case report

**DOI:** 10.1186/s13019-014-0183-8

**Published:** 2014-12-14

**Authors:** Hani Shennib, Bradley Bowles, Kelli Hickle

**Affiliations:** Arizona Heart Hospital, 1930 East Thomas Road, Phoenix, 85016 AZ USA; College of Medicine – Phoenix, University of Arizona, 550 East Van Buren Street, Phoenix, 85004 AZ USA

**Keywords:** IVC filter migration, IVC filter fracture, Bard Recovery, Pericardial effusion, Ventricular perforation

## Abstract

A 23 year old woman presented with sudden onset retrosternal chest pain following an attempt to move a heavy object from her vehicle. Multiple fractured struts of an inferior vena cava filter were identified in the distal right and left pulmonary artery branches, and in the free wall of the right ventricle. A small pericardial effusion was noted. Because of the depth of penetration into the right ventricle, it was perceived not to be amenable to endovascular retrieval. Over several days of observation, she continued to have progressive retrosternal and left shoulder pain. She underwent exploratory sternotomy and extraction of a strut that was partially protruding from the right ventricle and abrading the diaphragmatic pericardium. The patient recovered quite well and was discharged on the third postoperative day.

**Electronic supplementary material:**

The online version of this article (doi:10.1186/s13019-014-0183-8) contains supplementary material, which is available to authorized users.

## Background

Migration of fragments is a known complication of inferior vena cava (IVC) filter placement. Fragments that migrate to the right side of the heart can further lodge in the right ventricular wall or may migrate further into the pulmonary arteries. Right ventricular wall implantation can progress to penetration, hemopericardium, tamponade and possible death. Management of an IVC fragment which migrates into the right ventricle can be challenging. There are neither clear guidelines nor sufficient literature of how to best manage and treat such complex situations.

We present a case of IVC filter fracture and migration into the right ventricle which was treated with sternotomy and extraction of the fragment from its free wall.

## Case presentation

Our patient is a 23 year old Caucasian female who presented to the emergency department complaining of severe, sharp mid-chest pain that increased with inspiration. The patient stated that the pain started while she was lifting a stroller and stooping to prepare a vehicle seat for her four month old child.

On presentation, she was in obvious distress and complaining of chest pain. She denied shortness of breath or any other symptoms. She denied any prior symptoms. On examination, she was found to be tachycardic, her blood pressure was normal and review of all other systems was negative.

Her electrocardiogram showed sinus rhythm and was within normal limits. The first troponin was elevated at 0.40 and the second was 0.29. All other labs were within normal limits.

CT angiography revealed no evidence of pulmonary embolism; however, four tiny metal objects consistent with fractured struts from an IVC filter were identified in the heart, left and right pulmonary arteries, and upper abdomen.

Records obtained from another hospital revealed that a Bard Recovery IVC filter (Bard Peripheral Vascular, Tempe, Arizona) was placed in the course of treating a life-threatening multiple trauma from a motor vehicle accident eight years previously. The patient apparently was in coma for a month and was unaware of the existence of the IVC filter.

**Figure 1:**
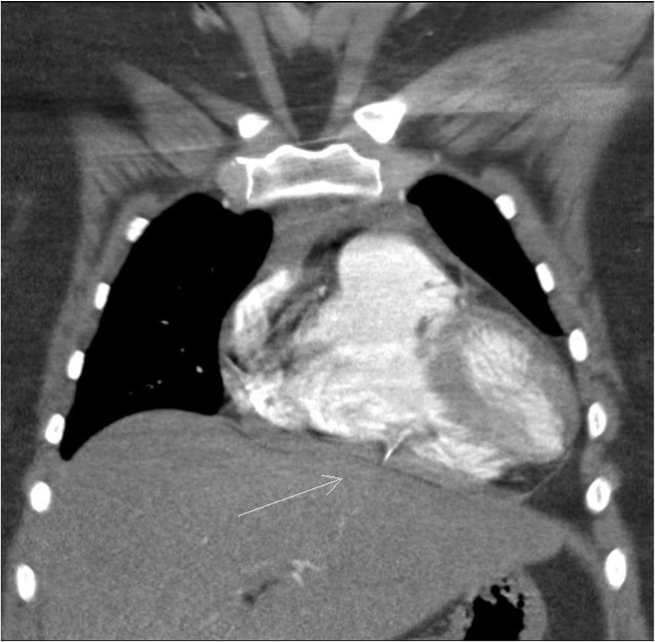
**Fractured strut embedded in the right ventricular free wall, protruding into the diaphragm.**

**Figure 2:**
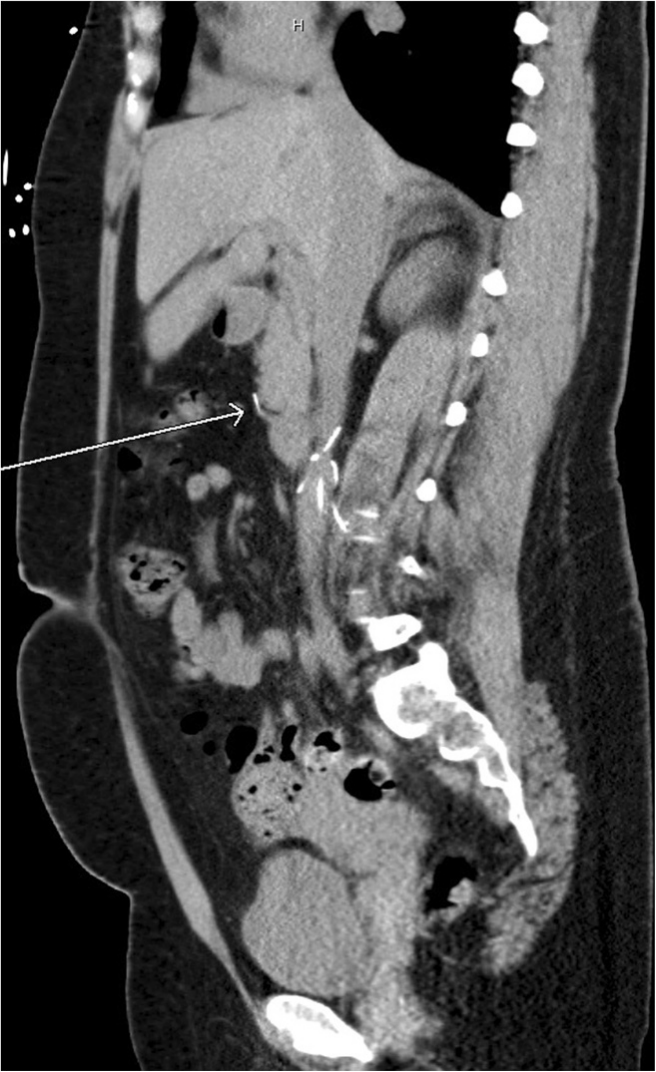
**Fractured strut migration near duodenal sweep.**

The first strut was protruding inferiorly through the free wall of the right ventricle and was associated with a small pericardial effusion (Figure 1). Two were found in the subsegmental branches of the pulmonary arteries of the left and right lungs. The fourth fragment was found outside the inferior vena cava along the duodenal sweep adjacent to the anterior-inferior margin of the uncinate process (Figure 2). A limb of the remaining structure of the IVC filter was observed to be penetrating into the adjacent abdominal aortic wall superior to the iliac bifurcation.

**Figure 3:**
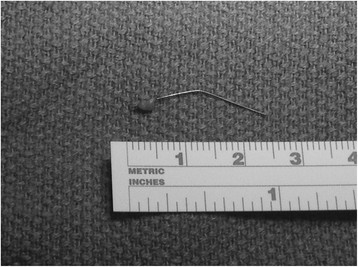
**Fractured strut retrieved from right ventricular free wall.**

Initial treatment was limited to in-hospital observation with pain management. However, the pain progressed and became intolerable with excruciating left shoulder pain over the ensuing 48 hours. A repeat CT revealed further penetration of the right ventricular strut through the ventricular wall and into the diaphragm with increasing pericardial effusion. Consequently, the patient was taken to the operating room where midline sternotomy and pericardiotomy was performed. Approximately 300 cc of blood was drained from the pericardial space. An intact, 2.5 cm metal strut was found protruding from the free wall of the right ventricle and was removed (Figure 3). The small laceration was repaired with a pledgeted 4–0 proline horizontal mattress suture. No cardiopulmonary bypass was needed.

The patient tolerated the procedure well and had immediate relief of her preoperative pain. She had an uneventful recovery and was discharged 3 days postoperatively. At a 3 month follow up appointment, the patient was doing well and had no symptoms. The patient subsequently had successful endovascular removal of the main fractured IVC filter.

## Conclusions

There appears to be an expanding use of IVC filters for prevention of pulmonary embolism worldwide [1]. Although IVC filters have been shown to be relatively safe and effective, significant complications have been reported. Fracture and migration is a recognized complication of IVC filter placement, however, patients may not be aware that a filter can fracture and migrate as in the presented case. Activities involving a sudden, intense increase in intra-abdominal pressure have been noted to be frequently associated with filter fracture and migration. Chest pain is the most common presentation. Migration of the complete filter or a fractured segment to the heart or lungs may also manifest with new acute shock and hemodynamic instability.

CT scans of the chest appear to be the most suited radiologic modality for identifying migrated fragments of an IVC filter within the chest. Correlation between chest pain and the location of migrated struts can assist in making management decisions. Increasing chest and shoulder pain with progression of pericardial effusion warrants intervention. Radiologic evaluation of the location and depth of penetration of the strut within the right ventricle may assist in determining if an endovascular or open surgical approach is more appropriate.

Our patient presented with chest pain that was initially controlled pharmacologically. She was hemodynamically stable; however, we observed increasing chest pain and progression of the pericardial effusion, which justified intervention. A less-invasive endovascular retrieval would have been preferred, but the fractured strut appeared to be advancing further into the ventricular free wall and diaphragm, making the retrieval more technically challenging. Because our patient was young and otherwise in good health, open surgery was chosen as a definitive method for the strut retrieval, cardiac defect repair and effusion drainage.

There is a paucity of studies addressing the optimal management of patients with migrating struts from fractured IVC filters. A review of the literature revealed only eight case reports [2]-[9] clearly documenting IVC filter fragment migration to the right ventricle. A variety of treatment options ranging from conservative pain management to endovascular or open surgical extraction of the migrating struts have been suggested. However, no clear recommendations have been proposed as to the optimal modality. Cardiovascular surgeons need to be aware of the increasing prevalence of IVC filters and the risk of fracture and migration to the right ventricle. Further studies and guidelines are needed to appropriately manage this complication.

## Consent

Informed consent was obtained from the patient for publication of this Case report and any accompanying images. A copy of the written consent is available for review by the Editor-in-Chief of this journal.

## Authors’ original submitted files for images

Below are the links to the authors’ original submitted files for images. Authors’ original file for figure 1 Authors’ original file for figure 2 Authors’ original file for figure 3

## Abbreviations

IVC: Inferior vena cava
