# Supplementary figures and images for: Migration of a fractured inferior vena cava filter strut to the right ventricle of the heart: a case report
Source: J Cardiothorac Surg. 2014 Dec 14;9:183. doi: 10.1186/s13019-014-0183-8 (PMC4299290; doi:10.1186/s13019-014-0183-8)

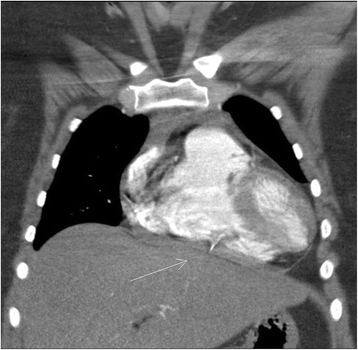

Supplement: Supplementary file 1 — Authors’ original file for figure 1 [file 13019_2014_183_MOESM1_ESM.gif]

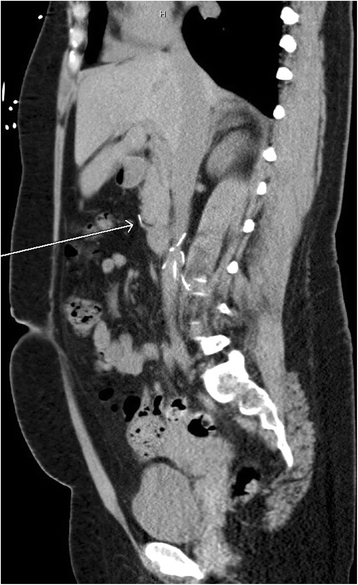

Supplement: Supplementary file 2 — Authors’ original file for figure 2 [file 13019_2014_183_MOESM2_ESM.gif]

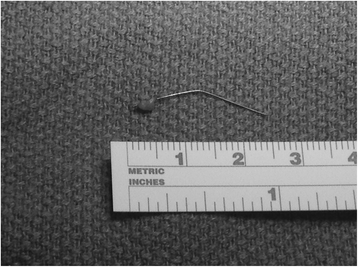

Supplement: Supplementary file 3 — Authors’ original file for figure 3 [file 13019_2014_183_MOESM3_ESM.gif]
